# Innovative intraoral cooling device better tolerated and equally effective as ice cooling

**DOI:** 10.1007/s00280-017-3434-2

**Published:** 2017-10-03

**Authors:** Java Walladbegi, Martin Gellerstedt, Anncarin Svanberg, Mats Jontell

**Affiliations:** 1 Department of Oral Medicine & Pathology, Institute of Odontology, The Sahlgrenska Academy, University of Gothenburg, Box 450, 405 30 Gothenburg, Sweden; 2 University West, School of Business, Economics and IT, University West, S-461 86 Trollhättan, Sweden; 3 Department of Hematology, Institute for Medical Sciences, Faculty of Medicine, Uppsala University Hospital, Uppsala University, 751 85 Uppsala, Sweden

**Keywords:** Cryotherapy, Tolerability, Healthy volunteers, Intra-oral cooling device, Oral mucositis, Myeloablative therapy

## Abstract

**Purpose:**

Most of the patients who receive myeloablative therapy prior to stem cell transplantation develop oral mucositis (OM). This adverse reaction manifests as oral mucosal erythema and ulcerations and may require high doses of morphine for pain alleviation. OM may also interfere with food intake and result in weight loss, a need for parenteral nutrition, and impaired quality of life. To date, there have been very few studies of evidence-based interventions for the prevention of OM. Cryotherapy, using ice chips, has been shown to reduce in an efficient manner the severity and extent of OM, although clinical applications are still limited due to several shortcomings, such as adverse tooth sensations, problems with infectious organisms in the water, nausea, and uneven cooling of the oral mucosa. The present proof-of-concept study was conducted to compare the tolerability, temperature reduction, and cooling distribution profiles of an intra-oral cooling device and ice chips in healthy volunteers who did not receive myeloablative treatment, and therefore, did not experience the symptoms of OM.

**Methods:**

Twenty healthy volunteers used the cooling device and ice chips for a maximum of 60 min each, using a cross-over design. The baseline and final temperatures were measured at eight intra-oral locations using an infra-red thermographic camera. The thermographic images were analysed using two digital software packages. A questionnaire was used to assess the tolerability levels of the two interventions.

**Results:**

The intra-oral cooling device was significantly better tolerated than the ice-chips (*p* = 0.0118). The two interventions were equally effective regarding temperature reduction and cooling distribution.

**Conclusions:**

The intra-oral cooling device shows superior tolerability in healthy volunteers. Furthermore, this study shows that temperature reduction and cooling distribution are achieved equally well using either method.

**Electronic supplementary material:**

The online version of this article (doi:10.1007/s00280-017-3434-2) contains supplementary material, which is available to authorized users.

## Introduction

A majority of the patients who undergo treatment for cancer that involves radiation and/or chemotherapy are at risk of side-effects, such as nausea/vomiting [1], diarrhoea [2], reduced salivary flow, infections, dysphagia, xerostomia, dental caries, osteoradionecrosis, and oral mucositis (OM), of which the latter is acknowledged as one of the most severe side-effects [3]. OM affects approximately 40% of patients who are treated with standard-dose chemotherapy, and up to 80% of those who receive high-dose chemotherapy [4, 5]. One of the most severe and distressing symptoms of OM is oral pain [6], although the symptoms of OM can also affect patient comfort, speech, nutritional status, and ability to tolerate medical treatment [7]. In addition, OM is associated with weight loss, parenteral feeding, impaired quality of life [8], and extended hospital visits [9], and it is considered to have adverse effects on socio-economical well-being [10].

A long-standing concern for patients with cancer of the blood or bone marrow (e.g., myeloma) who are scheduled to receive high doses of chemotherapy is the establishment of an effective and well-tolerated preventive treatment strategy to alleviate OM in conjunction with hematopoietic stem cell transplantation (HSCT). Current recommendations regarding the prevention of OM involve the use of recombinant human Keratinocyte Growth Factor-1 (Palifermin), low-level laser therapy (LLLT), and cryotherapy (CT) [11].

In the field of haematology/oncology, CT using ice chips before, during, and after chemotherapeutic drug infusion has proven to be an effective treatment modality for alleviating OM [12–15]. The presumed mechanism is vasoconstriction, which reduces the blood flow and thereby reduces tissue exposure to chemotherapeutic agents. Another hypothesis is that CT reduces the metabolic activity of the basal epithelial cells, resulting in lower absorption of the agent and reduced chemotherapy-induced damage [14].

Despite the fact that CT efficiently reduces OM in conjunction with HSCT, this cooling method can have adverse events, such as chills, headache, numbness/taste disturbance, and teeth sensations [16]. In addition, it is uncertain whether all parts of the oral cavity are cooled equally using this method. Moreover, a continuous supply of ice chips is needed during treatment sessions, and it is often the case that the water used to make the ice chips is of poor quality, creating a health risk [17]. To date, ice chips have been the only documented preventive cooling method available in clinical practice for these patients, and alternative cooling methods have not been investigated. To address this deficit, we have developed an innovative disposable cooling device (intra-oral cooling device; CD) that comprises an enclosed channel system with a continuously circulating hypothermic medium.

The main objectives of the present study were to compare in a randomised cross-over trial with healthy subjects, the tolerability, temperature reduction, and cooling distribution profiles of the intra-oral cooling device and ice chips.

## Subjects and methods

### Trial design

This was a prospective randomised cross-over trial to compare a new cooling device and ice chips as cryotherapies. The Research Randomizer software (https://www.randomizer.org/) was used to assign randomly the subjects to the order in which the two procedures were to be commenced. Half of the subjects started with the cooling device and crossed over to ice chips, while the other half of the subjects undertook the two procedures in the reverse order.

### Subjects

The study involved a total of 20 dental students, 17 women and 3 men (mean age, 23.9 years; range, 21–35 years), who were recruited between April 2016 and May 2016 from the Institute of Odontology, The Sahlgrenska Academy, University of Gothenburg, Gothenburg, Sweden. All the participants were healthy and had no medical conditions or used any drugs with substantial impact on the cardiovascular system. None of the participants had mucosal lesions, were smokers or users of oral tobacco products. The characteristics of the participants are listed in Table 1.

**Table 1:** Demographic characteristics of the study subjects

Characteristic	Mean	SD	Min	Max
Age (years)	23.9	3.2	21	35
Length (cm)	107.4	7.4	160	185
Weight (kg)	68.4	10.0	53	90
BMI	23.6	3.5	18.9	33.1

## Ethical approval

All the procedures performed in the studies involving human participants were approved by the local Ethical Review Board and in accordance with the 1964 Helsinki Declaration and its later amendments or comparable ethical standards.The study was approved by the local board of the Department of Oral Medicine and Pathology, Institute of Odontology, Sahlgrenska Academy, University of Gothenburg, Gothenburg, Sweden. The regional ethical review board in Gothenburg did not consider an ethical application necessary. Informed written consent was obtained by from all the participants.

## Tools and devices

### Cooling device

**Fig. 1:**
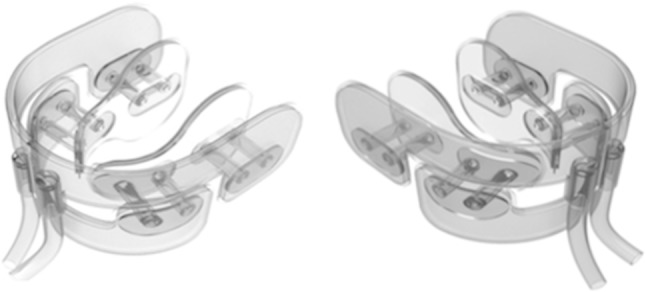
Schematic of the cooling device

The cooling device (Cooral™; Fig. 1) was provided by a Swedish medical technology company (BrainCool AB, Lund, Sweden). The device is composed of a soft plastic material with conduits for water, which is delivered via a portable cooling and thermostat unit (BrainCool). The unit, which produces water at temperatures that can be set between 6 and 22 °C, is connected to the intra-oral cooling device by tubes that allow a flow rate of 0.25 ml/min. A water temperature of 8 °C with a flow rate of 0.25 ml/min was used throughout the cooling procedure.

### Ice chips

Ice chips were produced in a commercial ice maker using tap water. Prior to use, the temperature of the ice chips was −0.5 °C. The ice chips were stored in a metal container at room temperature during the cooling procedure.

### Questionnaire

A questionnaire was applied that was specially designed by the authors for this study and that consisted of 15 questions, which were primarily aimed at evaluating the tolerability of the cooling methods for the subjects. The questionnaire also included questions regarding: the reasons why the cooling procedure was not completed or discontinued; and any adverse events experiences for each of the two cooling methods. There was also space to share other comments in running text. Prior to the study, all the questions and response alternatives were tested and discussed with an independent group of participants (*n* = 5). In this manner, the questionnaire was face-validated to ensure that the questions were interpreted as intended.

### Tools and software for image analysis

**Fig. 2:**
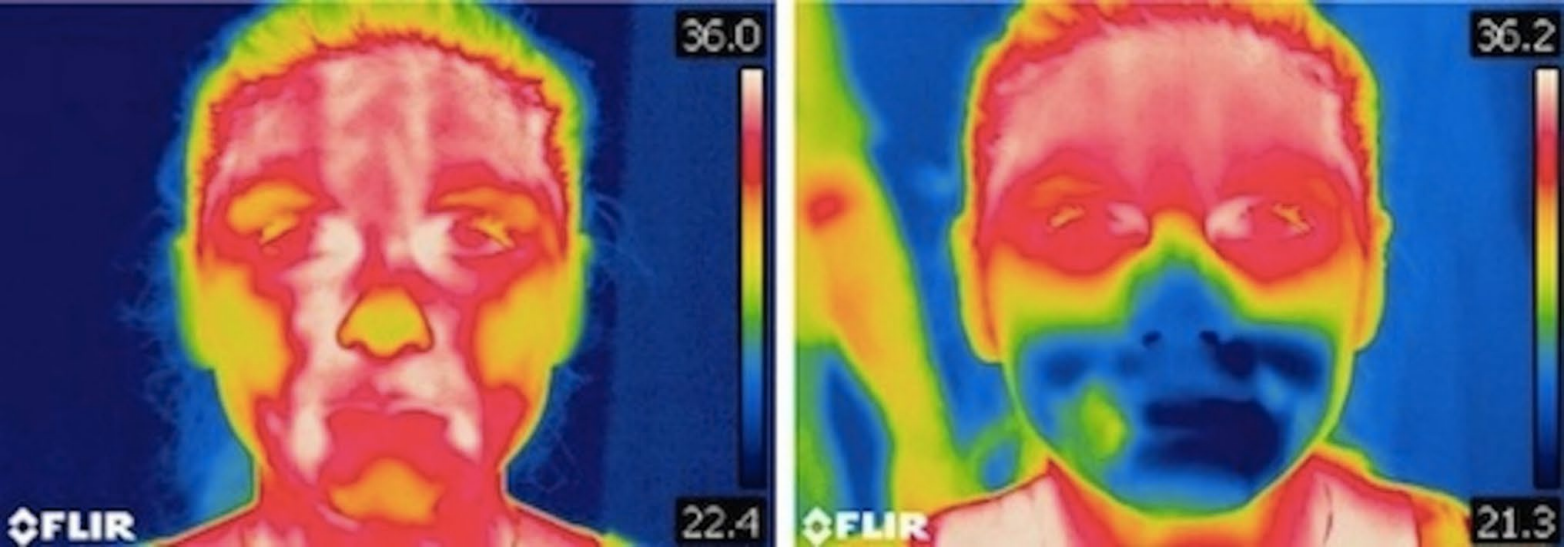
Images taken before and after cooling with the cooling device to illustrate the FLIR E60(bx) camera technique. The red colour indicates high temperatures and the blue colour indicates low temperatures

The FLIR E60(bx) (FLIR Systems Inc., Wilsonville, OR, USA) is an accurate thermographic camera with a level of resolution (320 × 240 pixels) that allows the detection of temperature differences of <0.05 °C (Fig. 2). The camera and the associated FLIR tools software were used to visualise and quantify changes in surface temperatures. For this study, the FLIR tools software was used to detect temperature reductions, and a freely available multi-research software tool (BioPix; http://www.biopix.se) was used to assess the cooling distribution by automatically quantifying the percentage of an image that corresponded to a specific temperature. The Omron M3 Comfort digital monitor (Omron, HigashiNoda, Osaka, Japan) was used to measure blood pressure and heart rate.

## Procedure and data collection

Eligible subjects were examined in a dental office (ambient temperature 22 °C) located at the Department of Oral Medicine and Pathology Institute of Odontology, The Sahlgrenska Academy, University of Gothenburg, Gothenburg, Sweden. Prior to inclusion, each individual was provided with detailed information and instructions regarding the usage of the two cooling methods, and written informed consent was obtained. The length (in centimetres) and weight (in kilograms) were measured and the BMI was calculated for each subject. Subjects were asked to complete a form to gather information about their medical history. The participants used the cooling device and ice chips for a maximum of 60 min in two separate sessions at least 24 h apart.

### Cryotherapy using the cooling device

The cooling device, which was available only in one size, was self-inserted under surveillance and adjusted by the subject until it felt comfortable. A staff member verified a good adaptation to the oral mucosa before the cooling was started.

### Cryotherapy using ice chips

Subjects inserted an ounce of ice chips and were asked to move the ice chips around in the mouth so as to cool as large a part of the oral mucosa as possible. When a melted ice slurry was obtained, the subject gargled for a few seconds before swallowing or spitting out the slurry. If the ice had melted completely another table spoon of ice was inserted immediately.

Prior to and immediately after cryotherapy, the temperature level at eight intra-oral locations (right buccal mucosa, left buccal mucosa, upper labial mucosa, lower labial mucosa, anterior and posterior dorsal tongue, anterior ventral tongue, and hard palate) were measured using the FLIR E60(bx) system. Blood pressure (systolic and diastolic) and heart rate were measured in the left arm with the subjects in a sitting position using the Omron M3 Comfort digital monitor. Following each cooling session, the subjects answered the questions regarding completed cooling in the questionnaire.

In total, 700 thermographic images were captured using FlIR E60(bx). The images were analysed by a blinded observer using the FLIR tools and BioPix software for assessments of temperature reduction and cooling distribution, respectively.

The maximum, minimum, and mean temperatures were recorded for each of the eight intra-oral locations before and after the cooling session, using the FLIR tools software. In addition to the temperature data, the software assigned a colour to each temperature, which was subsequently used in BioPix to assess cooling distribution. The analysis with BioPix was, however, only performed on the thermographic images captured after cooling, by calculating the percentage of an image that was covered by a specific colour, equivalent to or less than the mean temperature achieved from the previous temperature analysis at each location.

For the statistical analysis, the average value, taking into account the mean for each intra-oral location, was calculated and used both for temperature reduction and cooling distribution results.

## End-points

### Primary end-point

The primary end-point regarding tolerability was the extent to which an individual expressed a preference for one of the two methods by responding to the question: “Which of the two cooling methods did you tolerate better?”.

### Secondary end-points

The secondary end-points were: mean temperature reduction; and mean cooling distribution.

## Statistical analysis

With 20 participants, the power of the study for detecting a difference in preference of at least 80% (at least 80% of the subjects favour one of the two treatments) was 80%, given a significance level of 5%, using a two-sided sign test. The primary end-point, expressed by “Which of the two cooling methods did you tolerate better?”, was analysed using a two-sided sign test (McNemar’s test) and an exact 95% confidence interval. The secondary end-points of mean temperature reduction and mean cooling distribution were analysed using a paired samples *t*-test, with corresponding confidence intervals (95%). Systemic variables were analysed in the same manner. The quantitative variables were also analysed by ANOVA, including factors for treatment, sequence, period, and subject nested with sequence. The standard Pearson correlation was used for analysing associations between systemic variables and the secondary end-points, by cooling method. The associations between BMI and secondary end-points were analysed in the same manner. A significance level of 5% was used. The analysis of the primary variable was done using the SPSS ver. 23 statistical analysis software package (IBM, Armonk, NY, USA).

## Results

### Tolerability

The cooling device was preferred in comparison to ice chips as 16 out of 20 participants (80%) favoured this cooling method (*p* = 0.0118; 95% CI 0.563 to 0.943). Among the subjects who started with ice chips followed by the cooling device, nine out of ten preferred the cooling device, and for the subjects following the opposite procedure, seven out of ten preferred the cooling device, (*p* = 0.291).

A total of 40 cooling sessions (20 cooling device/20 ice chips) were conducted during the study and thirty-six sessions were completed. Thirty-four of the total cooling sessions, which accounted for 17/20 individuals, completed the entire time span of 60 min with both cooling methods. Two of the remaining three individuals completed one of the two methods each and two sessions were interrupted (one cooling device and one ice chips) because of discomfort. One of the three individuals interrupted two of the four sessions with both the cooling device and ice chips due to hypersalivation and nasal congestion. All of the subjects completed the questionnaires related to tolerability.

### Adverse events

The adverse events for each of the cooling methods are presented in Table 2. Cold (*n* = 12) and numbness (*n* = 11) were the most common adverse events reported for ice chips followed by teeth sensations (*n* = 8) and pain (*n* = 5). On the contrary, difficulties with swallowing (*n* = 15), rubbing discomfort (*n* = 12) and poor fit (n = 7) were the most common adverse events reported for the cooling device.

**Table 2:** Adverse events for the use by the subjects of ice chips or the cooling device

Adverse event	Ice chips (*n*)	Cooling device (*n*)
Cold	12	3
Numbness	11	3
Bad taste	3	1
Headache	2	0
Teeth sensations	8	2
Pain	5	3
Poor fit^a^	0	7
Nausea	4	1
Vomiting sensation	1	3
Difficulties in swallowing	0	15
Rubbing discomfort^a^	2^b^	12

^a^Alternative only available for the cooling device

^b^Reported as ‘other comments’

### Cooling effect, systemic variables and BMI

The cooling device showed equivalent properties as ice chips in terms of mean temperature reduction. As shown in Tables 3 and 4, the mean temperature reduction for ice chips was 8.08 °C while it was 7.91 °C for the cooling device, hence a mean difference of 0.17 °C (*p* = 0.795; 95% CI −1.18 to 1.52). The mean cooling distribution was 48.44% for ice chips and 47.33% for the cooling device, i.e., a mean difference of 1.11% (*p* = 0.457; 95% CI −1.97 to 4.21). Furthermore, we found no statistically significant mean differences in blood pressure or heart rate change. The systolic BP increased 1.10 mmHg in average for ice chips compared to a decrease of 0.50 mmHg for cooling device, i.e., a mean difference of −1.60 mmHg (*p* = 0.674; 95% CI −9.45 to 6.25). Corresponding figures for diastolic BP were average increase of 2.70 mmHg for ice chips, 0.55 mmHg for cooling device and hence a mean difference of 2.15 mmHg (*p* = 0.503; 95% CI −8.74 to 4.44). The average heart rate decreased in both groups 8.55 vs. 5.95 meaning that the mean difference was 2.60 beats per minute (*p* = 0.196; 95% CI −1.46 to 6.66). BMI did not have any statistically significant impact on temperature reduction or cooling distribution regardless of the method used.

**Table 3:** Mean temperature reductions and cooling distributions of the subjects who received ice chips or the cooling device

Subject	Temperature reduction (°C)		Cooling distribution (%)	
	Ice chips	Cooling device	Ice chips	Cooling device
1*	8.50	8.90	51.48	46.76
**2**	10.80	10.80	54.81	55.81
**3**	4.90	9.60	48.41	52.49
*4*	11.00	11.10	45.69	50.96
**5**	6.10	8.90	52.23	49.14
6*	7.90	6.30	48.06	43.33
*7*	6.40	6.60	53.58	39.44
*8*	10.80	9.00	50.29	43.45
*9*	9.10	6.90	43.66	50.59
*10*	10.60	9.20	46.63	50.59
*11*	10.70	8.80	45.28	49.01
*12*	8.10	5.90	45.76	49.30
**13**	9.40	9.50	46.13	48.00
**14**	3.10	8.70	47.35	45.45
*15*	10.30	8.90	49.28	49.68
*16*	9.20	9.40	43.58	52.05
**17**	7.00	7.60	48.23	41.36
18*	8.00	1.60	41.48	39.39
**19**	5.20	1.60	59.25	42.36
20*	4.40	8.80	47.69	47.41
Mean	8.08	7.91	48.44	47.33

The subjects marked in italics font started cooling with ice chips and the subjects marked in bold font started cooling with the cooling device. Subjects who tolerated ice chips better than the cooling device are marked with *

**Table 4:** Comparisons between Ice chips (Ice) and Cooling device (CD)

Variable	Ice (mean)	CD (mean)	Mean difference	*p* value	95% CI	
Temperature reduction (°C)	8.08	7.91	0.17	0.795	−1.18	1.52
Cooling distribution (%)	48.44	47.33	1.11	0.457	−1.97	4.21
Systolic BP change (mmHg)	−1.10	0.50	−1.60	0.674	−9.45	6.25
Diastolic BP change (mmHg)	−2.70	−0.55	−2.15	0.503	−8.74	4.44
Heart rate change (beats/min)	8.55	5.95	2.60	0.196	−1.46	6.66

The ANOVA gave no statistically significant sequence effects. There were, however, two significant period effects for temperature reduction and change in systolic blood pressure (Table 5). The significant period effect for temperature reduction is related to the fact that both treatments showed lower temperature reductions in the second period, as compared to the first period (Table 6). The change in systolic blood pressure was negative in the first period and positive in the second period for both treatments, which is related to the significant period effect (Table 6).

**Table 5:** Mean value for each measured variable, categorised by the order of use of the ice chips and cooling device and the period of use

Variable	Sequence	Period 1	Period 2
Temperature reduction (°C)	Ice–device	9.42	7.74
	Device–ice	8.07	6.73
Cooling distribution (%)	Ice–device	46.52	47.43
	Device–ice	47.21	50.36
Systolic BP change (mmHg)	Ice–device	−7.40	1.50
	Device–ice	−0.50	5.20
Diastolic BP change (mmHg)	Ice–device	−0.50	−0.50
	Device–ice	−0.60	−4.90
Heart rate change (beats/min)	Ice–device	8.30	5.40
	Device–ice	6.50	8.80

**Table 6:** Estimated differences between treatments and the *p* values for sequence and period effects, from ANOVA

Variable	Mean treatment difference (ICE-CD)	*p* value	Sequence effect *p* value	Period effect *p* value
Temperature reduction (°C)	0.17	0.764	0.199	0.014
Cooling distribution (%)	1.11	0.446	0.169	0.174
Systolic BP change (mmHg)	−1.60	0.648	0.222	0.048
Diastolic BP change (mmHg)	−2.15	0.509	0.501	0.509
Heart rate change (beats/min)	2.60	0.208	0.767	0.882

## Discussion

This study was conducted to compare the tolerability levels of an innovative cooling device and ice chips in healthy volunteers, as well as to investigate any adverse events. Overall, cooling was well tolerated, which is in accordance with a previous review article by Kadakia et al. [18]. However, 16 of the 20 subjects preferred the intra-oral cooling device over the ice chips. For those who favoured the ice chips, three individuals frequently habitually chewed on ice chips and one experienced the cooling device as being too large, which is an issue that will be addressed in the future by creating three different sizes of the device.

Adverse events related to cryotherapy have, to the best of our knowledge, not been given prominence in previous studies, and therefore, have not been carefully evaluated. In the present study, several adverse events were reported for each of the cooling methods, with coldness, numbness, and teeth sensations being more frequently perceived in the cooling sessions with ice chips. This is not surprising, since ice chips at −0.5 °C were used and they were in direct contact with the oral mucosa and the teeth. In contrast, the cooling device is an enclosed channel system with circulating water at 8 °C, so there is no direct contact between the cold liquid and the surrounding tissues. The reason for using a water temperature of 8 °C was to avoid the addition of an anti-freeze coolant. Difficulties related to swallowing, rubbing discomfort, and poor fit were the most frequently reported adverse events for the cooling device, and these complaints may reflect the fact that only one size of the cooling device was available for this study. However, a study using devices of different sizes needs to be conducted to test this hypothesis. Furthermore, poor fit and rubbing discomfort are of great importance as they may cause sensitivity and damage to the oral mucosa, which would potentially worsen the OM. Therefore, a pilot study needs to be conducted with various sizes of the device in patients who are receiving myeloablative treatment, together with an assessment of adverse events. This would ensure that the adverse events experienced in healthy volunteers will not result in damage to the oral mucosa and exacerbation of OM.

Pain and nausea were also reported as adverse events for both methods, although these adverse events were reported more frequently in the sessions using ice chips. The nausea may be due to the volumes of water swallowed by the subjects as a consequence of the ice chips melting in the mouth, and the experienced pain is attributable to the low temperature of the ice. Nausea caused by the cooling device might be related to over-extension in some patients.

Regarding the adverse events noted, there is a clear trend towards design-related problems with the cooling device, whereas cold sensations and numbness accounted for the majority of the problems experienced with the ice chips. One major advantage of the cooling device over ice chips is that there is the possibility to refine and improve the design to achieve even better comfort, which is not possible for the ice chips.

This study also compared the effects of the cooling device and ice chips on mean temperature reduction, mean cooling distribution, and the association between oral cooling and systemic variables. In addition, we investigated the potential correlation between BMI and differences in temperature reduction and cooling distribution. However, analyses of the thermographic images did not show any statistically significant differences between the two methods.

This outcome can be explained in two ways. First, the FLIR tools software and BioPix are not specifically designed for investigating intra-oral temperatures, leading to a risk of misinterpretation. Second, although the same conditions were used for both cooling methods, the temperature of the oral mucosa recovers quickly, which means that there is a narrow time-window to capture the images at all eight intra-oral locations. This could cause distortions and further complicate the image analysis.

Ultimately, since the study was not dimensioned for these secondary end-points, there is a possibility of type II errors, i.e., the study does not have a sufficient sample size to be able to detect a difference between the methods. However, the possibility that ice chips are superior to the new cooling device and that the study failed to show this due to being underpowered seem unlikely, as the descriptive statistics actually point in the opposite direction, indicating superiority for the cooling device. These results, however, indicate that the same levels of temperature reduction and cooling distribution are achieved using water at 8 °C in the cooling device and ice chips at −0.5 °C.

The ANOVA did show two significant period effects, although since both treatments were equally affected by the period effects, these did not affect the estimates of differences between the two methods.

The ANOVA did not show any significant effect of sequence, which means that no significant implications of unequal carry-over effects were observed. Naturally, this may be a type II error due to lack of power to detect such a difference, given the limited sample size in the present study. However, we believe that there are good grounds to assume that there are no carry-over effects due to the nature of the present study. First, there are no changes in the underlying health conditions, since we are studying healthy volunteers. Second, none of the treatment effects, e.g., adverse events, such as teeth sensation or rubbing discomfort, are likely to be carried over to the second period.

Little is known about oral cooling and its possible associations with systemic variables. Baydar et al. observed no local or systemic side-effects associated with the use of cryotherapy with ice chips [15]. In contrast, Svanberg et al. reported significantly higher (systolic) blood pressure levels following cryotherapy [19].

Oral cooling did not show any statistically significant impact on any of the systemic variables, and BMI had no impact on oral cooling, which is not surprising since the majority of the subjects were within the normal range for BMI. These results are of importance for future clinical studies, as potential systemic effects can hamper medical rehabilitation after chemotherapy. However, further studies are needed to determine if there are genuine systemic effects following oral cryotherapy, particularly in patients who are undergoing myeloablative therapy. Furthermore, the same cooling capacity can be expected in subjects regardless of their BMI values.

The prospective randomised cross-over design, which allows all the subjects to test and evaluate both cooling methods, combined with blinded analyses of the thermographic images, provides reliable data and strengthens the impact of the present study. Another advantage is that subjective and objective parameters, as well as systemic associations were evaluated.

A limitation of the present study is that it was conducted on healthy dental students, which could have influenced the results, as they knew that the cooling device was a novel cooling method compared to ice chips. The cooling device might be tolerated differently by patients in clinical practice when used in cooling sessions that are longer than 60 min.

## Conclusion

This study demonstrates that the cooling device is superior to ice chips in terms of its tolerability for the subjects tested. Furthermore, this study shows that the levels of temperature reduction and cooling distribution achieved using these two cooling methods are equivalent. The next step in this research will be to evaluate the cooling device in patients who are receiving myeloablative therapy prior to stem cell transplantation.

## Electronic supplementary material

Below is the link to the electronic supplementary material.

Supplementary material 1 (DOCX 74 KB)

Supplementary material 2 (JPG 41 KB)
