# Supplementary material for: Innovative intraoral cooling device better tolerated and equally effective as ice cooling
Source: Cancer Chemother Pharmacol. 2017 Oct 3;80(5):965–72. doi: 10.1007/s00280-017-3434-2 (PMC5676821; doi:10.1007/s00280-017-3434-2)
Supplement: Supplementary file 1 — Supplementary material 1 (DOCX 74 KB) [file 280_2017_3434_MOESM1_ESM.docx]

**Questionnaire about Cooral™ cooling**

1. Did you manage to have Cooral™ in your mouth the whole cooling time?

Yes (skip to question 4)

No

2. Roughly how long did you have Cooral™ in your mouth?

1-20 minutes

21-30 minutes

31-40 minutes

41-50 minutes

51-59 minutes

3. Which of the following was the reason? Mark the letter or letters.

A  I got cold

B  I became numb

C  It tasted bad

D  I got a headache

E  Shooting pains in my teeth

F  My mouth got sore

G  Poor fit

H  I felt nauseous

I  I felt I needed to vomit

J  It was difficult to swallow

K  Rubbing discomfort

L  Other.........................................

4. Was it unpleasant to have Cooral™ in your mouth?

No, not at all (skip to question 6)

No, hardly at all

Yes, a little

Yes, very much so

5. If you experienced some form of discomfort, in what way was it unpleasant? (several alternatives may be chosen)

A  I got cold

B  I became numb

C  It tasted bad

D  I got a headache

E  Shooting pains in my teeth

F  My mouth got sore

G  Poor fit

H  I felt nauseous

I  I felt I needed to vomit

J  It was difficult to swallow

K  Rubbing discomfort

L  Other.........................................

6. Did Cooral™ limit your ability to do something else during the time?

No, not at all

No, not very much

Yes, a little

Yes, very much so

7. Other viewpoints..................................................................................

**Questionnaire about cooling with ice**

1. Did you manage to have the ice in your mouth the whole cooling time?

Yes (skip to question 4)

No

2. Roughly how long did you have ice in your mouth?

1-20 minutes

21-30 minutes

31-40 minutes

41-50 minutes

51-59 minutes

3. Which of the following was the reason? Mark the letter or letters.

A  I got cold

B  I became numb

C  It tasted bad

D  I got a headache

E  Shooting pains in my teeth

F  My mouth got sore

G  I felt nauseous

H  I felt I needed to vomit

I  It was difficult to swallow

J  Other.........................................

4. Was it unpleasant to have the ice in your mouth?

No, not at all (skip to question 6)

No, hardly at all

Yes, a little

Yes, very much so

5. If you experienced some form of discomfort, in what way was it unpleasant? (several alternatives may be chosen)

A  I got cold

B  I became numb

C  It tasted bad

D  I got a headache

E  Shooting pains in my teeth

F  My mouth got sore

G  I felt nauseous

H  I felt I needed to vomit

I  It was difficult to swallow

J  Other.........................................

6. Did the ice limit your ability to do something else during the time?

No, not at all

No, not very much

Yes, a little

Yes, very much so

7. Other viewpoints..................................................................................

**Final question**

You have tested cooling of the oral mucosa with ice and cooling device. Which of the cooling methods did you tolerate better?

1. Ice much better
2. Ice better
3. Ice slightly better
4. No method better
5. Cooling-device slightly better
6. Cooling-device better
7. Cooling-device much better
