# Supplementary figures and images for: Innovative intraoral cooling device better tolerated and equally effective as ice cooling
Source: Cancer Chemother Pharmacol. 2017 Oct 3;80(5):965–72. doi: 10.1007/s00280-017-3434-2 (PMC5676821; doi:10.1007/s00280-017-3434-2)

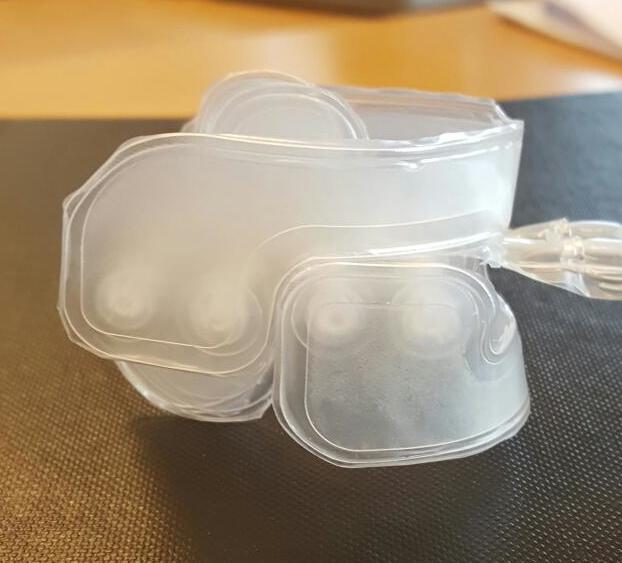

Supplement: Supplementary file 2 — Supplementary material 2 (JPG 41 KB) [file 280_2017_3434_MOESM2_ESM.jpg]
